# Supplementary material for: Towards Uncovering the Role of Incomplete Penetrance in Maculopathies through Sequencing of 105 Disease-Associated Genes
Source: Biomolecules. 2024 Mar 19;14(3):367. doi: 10.3390/biom14030367 (PMC10967834; doi:10.3390/biom14030367)
Supplement: Supplementary file 1 [file biomolecules-14-00367-s001.zip › Figure S1.pdf]

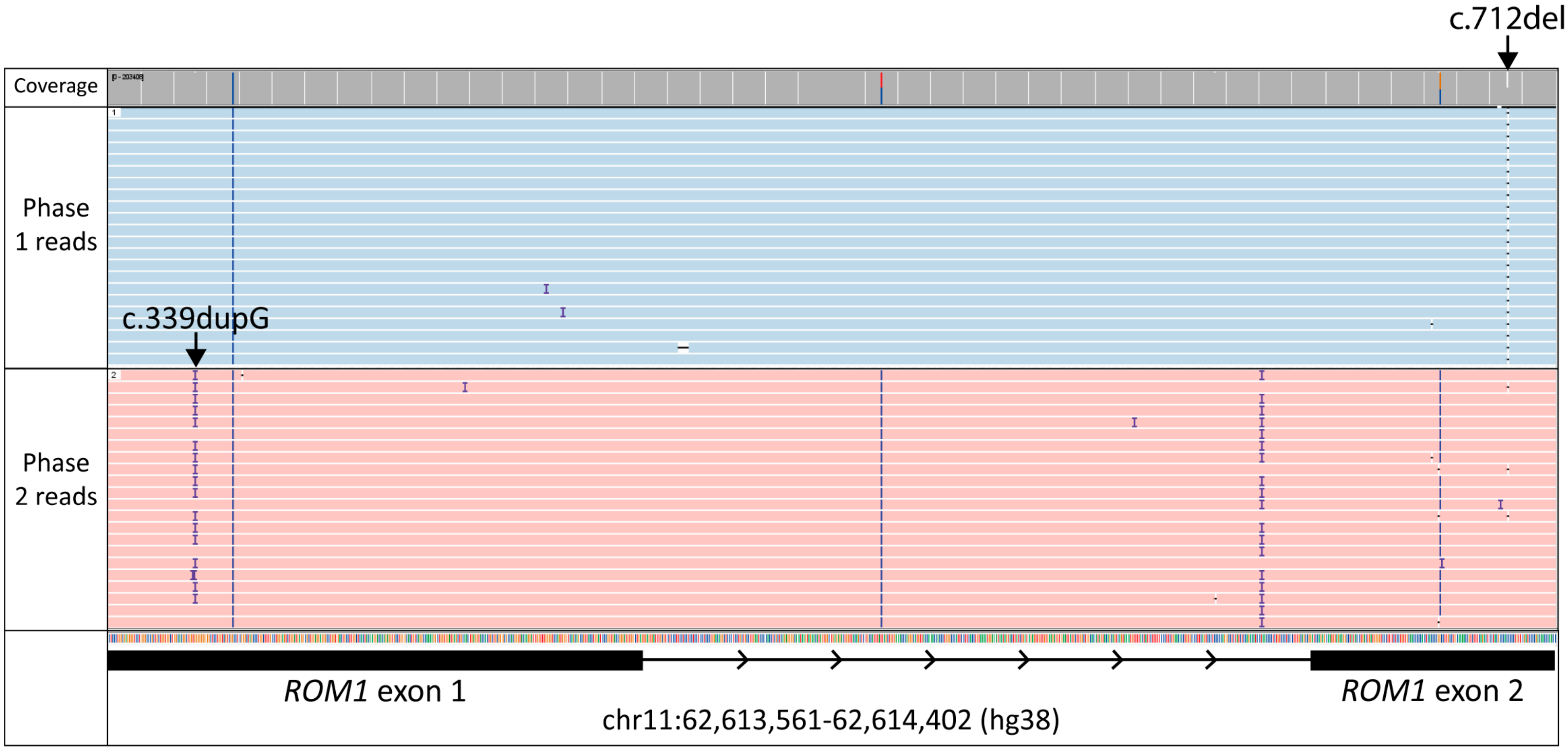

Figure S1: Variant phasing of *ROM1* variants using PacBio long-read amplicon sequencing. Primers were designed to amplify 1.2 Kb across *ROM1* variants c.339dupG and c.712del in proband 079830 and long-read amplicon sequencing was performed using PacBio. HiFi reads were visualized in IGV and grouped by phase to reveal that both variants are in *trans*.
