# Supplementary material for: Towards Uncovering the Role of Incomplete Penetrance in Maculopathies through Sequencing of 105 Disease-Associated Genes
Source: Biomolecules. 2024 Mar 19;14(3):367. doi: 10.3390/biom14030367 (PMC10967834; doi:10.3390/biom14030367)
Supplement: Supplementary file 1 [file biomolecules-14-00367-s001.zip › Supplementary Table S1-Oligonucleotides used throughout the study.pdf]

Supplementary Table S1: Oligonucleotides used throughout the study.

A. Primers with gateway cloning tags for *ABCA4* intron 49 minigene creation.

Minigene	Variant	Sequence (5’-3’)
BA39	ABCA4_c.6817-679C>G_Fwd	GGGGACAAGTTTGTACAAAAAAGCAGGCTTCCATTAAGCAGAGGGAGGCAC
	ABCA4_c.6817-679C>G_Rev	GGGGACCACTTTGTACAAGAAAGCTGGGTGCTGAGGGCACTGTGATTCTG

B. Site-directed mutagenesis primers used to introduce variants in wild-type entry clones for *ABCA4* midigene assays.

WT fragment	DNA variant_primer orientation	Mutagenesis primer sequences (5'-3')
BA7	ABCA4_c.1451A>G_mut_Fwd	CCCGAGGGCCCCTGTAGAGGAAGTTTAGGATGG
	ABCA4_c.1451A>G_mut_Rev	CCATCCTAAACTTCCTCTACAGGGGCCCTCGGG
BA16	ABCA4_c.3329-124G>T_Fwd	AGGAGTTAACTAAAAAAAAAAACACAAGGGAACTAATTCAGCAGCA
	ABCA4_c.3329-124G>T_Rev	TGCTGCTGAATTAGTTCCCTTGTGTTTTTTTTTTTAGTTAACTCCT

C. Primers used for reverse transcription-PCR analysis for *ABCA4* midigene assays.

BA_ID	Primer sequences (5’-3’)	Genomic position (hg19)
RHO_exon3_forward	CGGAGGTCAACAACGAGTCT	chr3:129251150
ABCA4_exon9	TTTGCTGATGGGAAAAATCC	chr1:94544941
RHO_exon5_forward	ATCTGCTGCGGCAAGAAC	chr3:129252475
RHO_exon5_reverse	AGGTGTAGGGGATGGGAGAC	chr3:129252614
ACTB_exon3_forward	ACTGGGACGACATGGAGAAG	chr7:5568922
ACTB_exon4_reverse	TCTCAGCTGTGGTGGTGAAG	chr7:5568099

D. Primers used for *ROM1* variant phasing.

BA_ID	Primer sequences (5’-3’)	Genomic position (hg19)
ROM1_Ex1_forward	GTGGCGTCATCCTCCTCTG	chr11: 62380848
ROM1_Int2_reverse	GCAAGGGGAAAGAGTCAGGG	chr11:62382062
