# Supplementary figures and images for: Towards Uncovering the Role of Incomplete Penetrance in Maculopathies through Sequencing of 105 Disease-Associated Genes
Source: Biomolecules. 2024 Mar 19;14(3):367. doi: 10.3390/biom14030367 (PMC10967834; doi:10.3390/biom14030367)

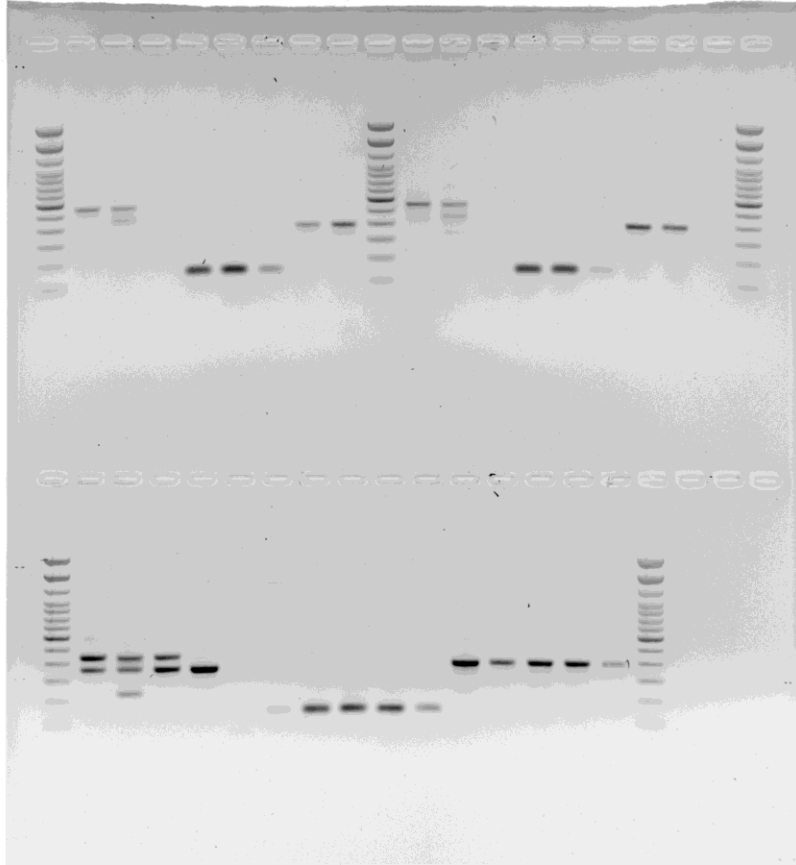

RT-PCR *ABCA4* c.1451A>G original gel image

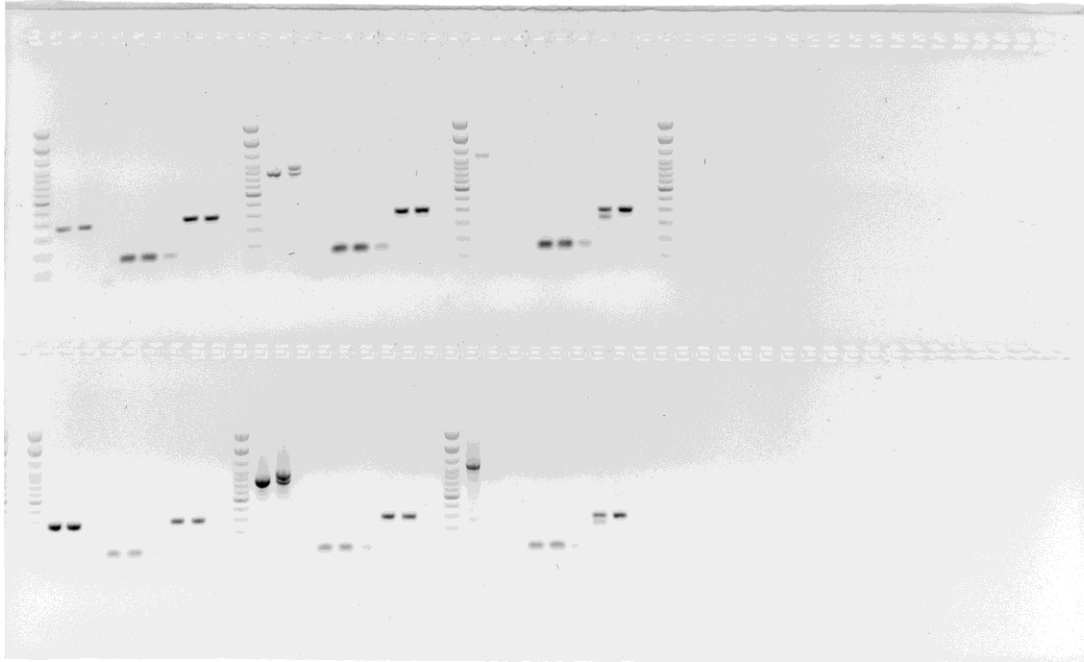

RT-PCR *ABCA4* c.6817-679C>G and c.3329-124G>T original gel image

Supplement: Supplementary file 1 [file biomolecules-14-00367-s001.zip › Supplementary File S1.pdf]
